# A Manual of Surgery

**Published:** 1886-06

**Authors:** 


*A Manual of Surgery.* In three volumes. In Treatises by various authors. Edited by FREDERICK TREVES, F.R.C.S. London: CASSELL & Co. 1886.

After carefully reading and, in the cases of not a few portions, re-reading this work, we have come to the conclusion that it is, for the student, the best work on English Surgery in existence. It is no disparagement to pass the trite remark, that "the articles being contributed by so many writers, are of unequal merit"; but it is more than can be said of any similar work by a single author, that none of the articles are weak or commonplace; that the great majority are excellent; and that not a few of them are models of their kind.

In the space at our disposal it is impossible to give even the most meagre summary of the various articles. A good many of them have been contributed by men whose authority on their subjects cannot be surpassed. Such are those by Hutchinson on Syphilis, Butlin on Tumours, Furneaux Jordan on Shock, Harrison Cripps on the Rectum, Henry Morris on the Abdomen and the Kidney, and Whitehead on the Mouth and Throat. Some of the very best articles are written by men less widely known: among these, we would select for special commendation those by Pearce Gould on Diseases of the Blood Vessels.

The *Manual* is thoroughly up to date; and in this respect it is superior to any manual, system, or cyclopædia with which we are acquainted. Crotchets, statistics, and bibliographies are conspicuous by their absence; pure science, ascertained fact, and the matured decisions of men competent to decide, form the matter of the work.

We confess that we should have preferred to possess the *Manual* bound in one "handsome octavo volume," index, contents, and all complete, within one set of quiet boards, to this little trinity of glaring red.

